# Copper Complexes as Anticancer Agents Targeting Topoisomerases I and II

**DOI:** 10.3390/cancers12102863

**Published:** 2020-10-05

**Authors:** Caroline Molinaro, Alain Martoriati, Lydie Pelinski, Katia Cailliau

**Affiliations:** 1 Univ. Lille, CNRS, UMR 8576-UGSF-Unité de Glycobiologie Structurale et Fonctionnelle, F-59000 Lille, France; caroline.molinaro@univ-lille.fr (C.M.); alain.martoriati@univ-lille.fr (A.M.); 2 Univ. Lille, CNRS, Centrale Lille, Univ. Artois, UMR 8181-UCCS-Unité de Catalyse et Chimie du Solide, F-59000 Lille, France; lydie.pelinski@univ-lille.fr

**Keywords:** copper complexes, topoisomerase inhibitor, DNA damage response, cell cycle, cell death, chemotherapy

## Abstract

**Simple Summary:**

Organometallics, such as copper compounds, are cancer chemotherapeutics used alone or in combination with other drugs. One small group of copper complexes exerts an effective inhibitory action on topoisomerases, which participate in the regulation of DNA topology. Copper complexes of topoisomerase inhibitors work by different molecular mechanisms that have repercussions on the cell cycle checkpoints and death effectors. The expansion of this family of highly active anticancer drugs and their use in combination with other emerging cancer therapies opens new avenues for the treatment of cancers.

**Abstract:**

Organometallics, such as copper compounds, are cancer chemotherapeutics used alone or in combination with other drugs. One small group of copper complexes exerts an effective inhibitory action on topoisomerases, which participate in the regulation of DNA topology. Copper complexes inhibitors of topoisomerases 1 and 2 work by different molecular mechanisms, analyzed herein. They allow genesis of DNA breaks after the formation of a ternary complex, or act in a catalytic mode, often display DNA intercalative properties and ROS production, and sometimes display dual effects. These amplified actions have repercussions on the cell cycle checkpoints and death effectors. Copper complexes of topoisomerase inhibitors are analyzed in a broader synthetic view and in the context of cancer cell mutations. Finally, new emerging treatment aspects are depicted to encourage the expansion of this family of highly active anticancer drugs and to expend their use in clinical trials and future cancer therapy.

## 1. Introduction

**Figure 1:**
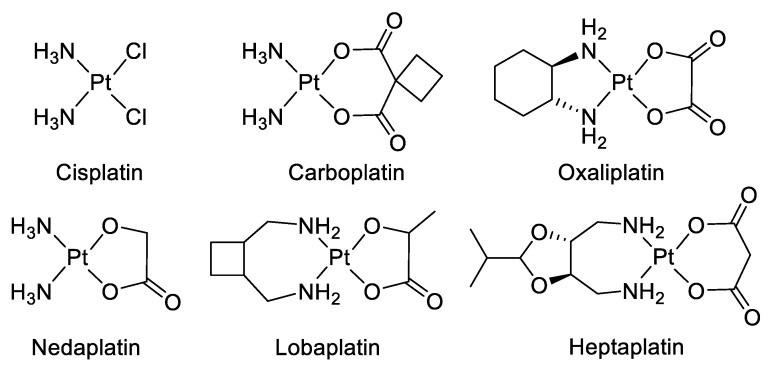
Platinum (II) complexes.

Chemotherapy is a systemic treatment proposed to patients suffering from cancer. It is often a complementary approach to surgery or radiotherapy. The discovery of platinum’s inhibitory effect on tumor cell growth in the 1960s [1] was a milestone for anticancer drug application in medicine [2]. Platinum (II) sets at the center of the squared planar structure of cisplatin and is coordinated with two chlorides and two ammonia molecules in a cis configuration. Cisplatin and its derivative drugs (carboplatin of second generation and oxaliplatin of third generation) are used worldwide in clinical applications and several other platinum analogs (lobaplatin, nedaplatin, and heptaplatin) are approved in several countries (Figure 1) [3,4]. However, serious side effects including toxicities on the kidney, heart, ear, and liver, decrease in immunity, hemorrhage, and gastrointestinal disorders limit the use of platinum derivatives [5,6,7]. The appearance of drug resistances, issuing from acquired or intrinsic multiple genetic and epigenetic changes, has also limited the clinical use of platinum-derived drugs [8]. Platinum-based treatment efficiency is challenged by cross-resistance and multiple changes including a decreased accumulation of the drug, a reduction in DNA–drug adducts, a modification in cell survival gene expression, an alteration of DNA damage repair mechanisms, modifications of transporters, protein trafficking, and altered cell metabolism [9,10,11,12,13,14].

To circumvent drug resistance, a possible approach consists of designing and developing new therapeutic metal-based anticancer drugs [15,16,17,18,19,20,21]. Several transition metals from the d-block of the periodic table (groups 3 to 12) and particularly essential trace metals [15,22,23], such as copper [24,25,26,27,28,29], are useful for the implementation of metal-based complexes in anticancer therapies. Copper plays central roles in various cellular processes being an essential micronutrient and an important cofactor for several metalloenzymes involved in mitochondrial metabolism (cytochrome c oxidase), or cellular radical detoxification against reactive oxygen species (ROS) (superoxide dismutase) [30]. Copper is essential for angiogenesis, proliferation, and migration of endothelial cells [31,32,33]. Elevated copper favors tumor growth and metastasis. It is detected in several brain [34], breast [35], colon, prostate [36], and lung [37] tumors and serves as an indicator of the course of the disease [38]. The differences in tumor cells’ responses to copper compared to normal cells laid the foundation of copper complexes’ (CuC) evolution as anticancer agents. Numerous developed CuC contain different sets of N, S, or O ligands and demonstrate high cytotoxicity and efficient antitumor activity [25]. Different mechanisms are involved in copper drugs’ anticancer effect. They act as chelators, and interact with and sequester endogenous copper, reducing its availability for tumor growth and angiogenesis [39]. On the contrary, ionophores trigger intracellular copper accumulation, cytotoxicity, and activate apoptosis inhibitor factor (XIAP) [24,40,41,42,43,44,45,46]. Other CuC are proteasome inhibitors [47,48]. Several CuC are actually on clinical trials: a number of copper/disulfiram-based drug combinations for therapy and as diagnostic tools (metastatic breast cancer and germ cell tumor), several casiopeínas compounds and elesclomol (leukemia), and thiosemicarbazone-based copper complexes labeled with a radioactive isotope for positron emission tomography imaging of hypoxia (in head and neck cancers) [49].

The cisplatin DNA-targeting principle of action also conditioned the development of anticancer copper-based drugs [4,23,50]. Antitumor activities of copper-based drugs are based on the interactive properties of both copper and the ligand. Copper toxicity results from its redox capacities (Cu(I) and Cu(II) redox states’ interconversion in oxidation–reduction cycles), the property to displace other ions from the enzyme binding sites, a high DNA binding affinity, and the ability to promote DNA breaks [28,51]. In most cases, copper modifies the backbone of the complexed ligand and grants better DNA affinity, specificity, and stability [52]. Copper derivatives can interact with DNA without the formation of covalent adducts. The noncovalent interactions with DNA include binding along with the major or the minor DNA grooves, intercalation, or electrostatic binding. Some copper-based drugs generate reactive oxygen species (ROS) that overwhelm cellular antioxidant defenses to produce oxidative damages in the cytoplasm, mitochondria, and DNA [53]. An important class of CuC, actually on focus for chemotherapy, inhibits topoisomerases (Top) 1 and 2, resulting in severe DNA damages, cell cycle arrest, and death [40,54,55,56,57]. Chemotherapeutics that target Top as poisons convert a transient DNA-enzyme complex into lethal DNA breaks [58,59,60,61,62]. However, topoisomerase inhibitors’ activity and their multifaceted binding modes to DNA, the effects, and the modulations they produce on the control of cancer cell division necessitate better understanding to optimize their efficiency.

This review focuses on CuC targeting human Top1 and Top2, the molecular mechanism of induced DNA damages, cell cycle arrest, programmed cell death responses, and emerging research strategies.

## 2. Copper Complexes as Topoisomerases Inhibitors

DNA topoisomerases have been molecular targets for anticancer agents since their discovery in 1971 [63]. Topoisomerases regulate DNA winding and play essential functions in DNA replication and transcription [59,64]. Topoisomerase 1 (Top1) creates transient single-DNA nicks, while topoisomerases 2 (Top2α and Top2β) produce transient double-stranded DNA breaks. Both nuclear Top1 and Top2 are important targets for cancer chemotherapy, and Top inhibitors are used in therapeutic protocols [65,66,67]. Top inhibitors are classified into two groups: poisons and catalytic inhibitors. Top poisons (or interfacial poisons) stabilize the reversible cleavage complex formed between Top and DNA and form a ternary complex. Top2 catalytic inhibitors can prevent DNA strands cleavage through inhibition of the ATPase activity (novobiocin, merbarone), by impeding ATP hydrolysis to block Top dissociation from the DNA (ICRF-193), or by DNA intercalation at the Top fixation site (aclarubicinet) see [68]. In all cases, inhibitors convert the indispensable nuclear Top enzyme into a killing tool.

Top inhibitors’ activity increases upon complexation with copper ion. Top1, Top2, or Top1/2 inhibitors synthesized in the form of copper complexes (CuC) are mostly mononuclear Cu(II) complexes associated with a variety of ligands (Table 1). Different strategies are currently proposed to design and develop Top inhibitory agents based on ligands’ properties [69]. If both Top1 and Top2 inhibitors CuC primarily target DNA by a direct interaction through intercalation or cleavage, their antiproliferative activity is reinforced by ROS production and other molecular targets (Table 1) [25,52].

### 2.1. CuC Top1 Inhibitors

**Figure 2:**
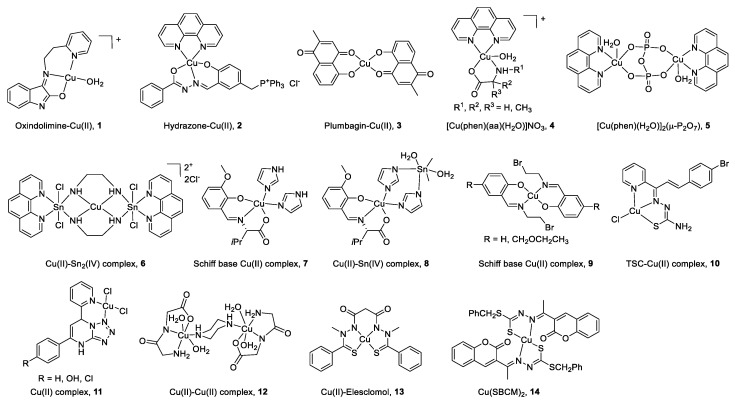
Structure of Cu(II) complexes as Top1 inhibitors.

All the structures of CuC Top1 inhibitors are reported in Figure 2 and the main characteristics in Table 1. Oxindolimine-Cu(II) Top1 inhibitors such as **1** are planar copper compounds [70] that do not permit enzyme-DNA complex formation [71,72,73]. Besides, they produce ROS [70]. Cu(II) derivative complexes of the hydrazone ligand with triphenylphosphonium moiety **2** can bind DNA and the Top enzyme [74]. Plumbagin-Cu(II) **3** selectively intercalates into DNA [75]. The latter compound [75] and the phenanthroline-Cu(II) complexes modulated by amino acids **4 [76]** can induce cancer cell apoptosis via mitochondrial signaling. Copper pyrophosphate-bridged binuclear complex **5** interacts with DNA, and based on the redox chemistry of copper, induces significant oxidative stress in cancer cell lines [77].

In the heterobimetallic Cu(II)-Sn_2_(IV) (copper/tin) complex **6**, the planar phenanthroline heterocyclic ring approaches the Top−DNA complex Cu(II)-Sn_2_(IV) toward the DNA cleavage site and forms a stable complex with Top1 [78,79]. Other Cu(II)-Sn_2_(IV) analogs induce apoptosis [80]. Chiral monometallic or heterobimetallic complexes **7** and **8** with tridentate chiral Schiff base–ONO-ligand are DNA groove binders and produce ROS [81,82].

Salicylidene-Cu(II) derivative **9** of 2-[2-bromoethyliminomethyl] phenol [83,84] is a bifunctional drug that inhibits both cancer cell growth and metastasis.

Chalcone-derived thiosemicarbazone (TSC) Cu(II) complex **10** prevents the DNA cleavage step of the Top1 catalytic cycle and DNA relegation [85].

Tetrazolo[1,5-*a*]pyrimidine-based Cu(II) complexes **11** have a square planar geometry, and despite their high capability to inhibit Top1, interact with CDK for **11** [86] and VEGF receptors for an analog of **11** [87]. Binuclear Cu(II) dipeptide piperazine-bridged complex **12** recognizes specific sequences in the DNA, oxidatively cleaves DNA, and displays superoxide dismutase (SOD) activity [88].

Derived from elesclomol (in clinical trials: phase 3 against melanoma and randomized phases 2 and 3 for the treatment of a variety of other cancers), the elesclomol-Cu(II) complex **13** inhibits Top1 and induces apoptosis in cancer cells [89].

As recently studied, Cu(II)(SBCM)_2_ **14** derived from *S*-benzyldithiocarbazate and 3-acetylcoumarin intercalates into DNA, induces ROS production, and has an antiproliferative activity in breast cancer lines [90,91].

### 2.2. CuC Top2α Inhibitors

Due to its cell cycle phase dependence and its high expression in proliferating cells, the Top2α isoform is primarily targeted by copper complexes (CuC), whereas Top2β remains unchanged during the course of the cell cycle [66]. Another reason to limit the clinical application of Top2β inhibitors is the strong unwanted side effects produced (secondary leukemia, myelodysplastic syndrome (MDS), and cardiac toxicity [92,93]).

**Figure 3:**
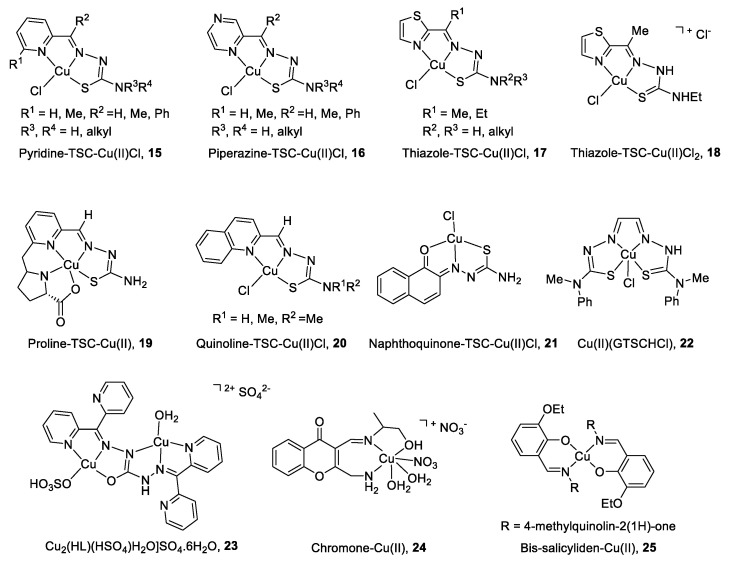
Structure of Cu(II) complexes as Top2 inhibitors.

The main characteristics and structures of CuC Top2 inhibitors are reported in Figure 3 and Table 1. Several α-(*N*)-heterocyclic thiosemicarbazone (TSC) CuC [94,95] present a greater inhibitory effect on Top2α than corresponding TSC ligands alone [96,97] due to a square planar structure around the Cu(II) ion. A specific subset of pyridine-TSC CuC **15** inhibits Top2α [98] acting as ATP hydrolysis inhibitors in a non-competitive mode [94,99,100]. Another pyridine-TSC CuC inhibits Top2β [100]. Molecular modeling supports the binding of the complexes near but outside the ATP binding pocket in communication with the DNA cleavage/ligation site of Top2. Piperazine-TSCs based CuC **16** inhibit Top2α [101,102] by a strong interaction with the ATP-binding pocket residues [99] without ROS production [102]. Thiazole-TSC CuC **17** and **18** are Top2α catalytic inhibitors [103,104] or poisons [105]. The highly water-soluble proline-TSC CuC series **19** inhibit Top2α and cell proliferation [106]. Quinoline-TSC CuC **20** interact with the DNA phosphate group preventing relegation. The presence of two methyl groups on the terminal nitrogen is responsible for high activity and confers a cationic nature responsible for easier passive access into the cell [107].

Non-heterocycle naphthoquinone-TSC CuC **21** [108] and bis-TSC CuC **22** [109] are Top2α inhibitors acting as poisons [109]; they induce apoptosis in various human cancer cell lines and delay colorectal growth of carcinoma xenografts in mice [109]. Carbohydrazone CuC **23** [110] is a Top2α inhibitor that binds DNA, induces apoptosis, and reduces mice xenograft (83% after a treatment of 2 mg/kg). Chiral chromone Cu(II)/Zn(II) **24** [111] revealed catalytic inhibition of Top2α with DNA binding in the major groove. Quinolinone CuC **25** [112] inhibit Top2α and DNA synthesis without DNA intercalation and are only minimized PGP (P-glycoprotein efflux transporter) substrates.

### 2.3. CuC Dual Top1/Top2α Inhibitors

**Figure 4:**
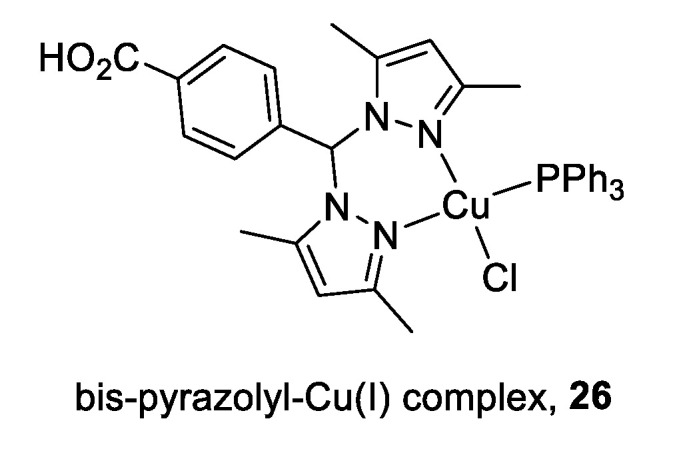
Structure of Cu(I) complex as a Top1/2α dual inhibitor.

Heteroleptic Cu(I) complexes of the bis-pyrazolyl carboxylate ligand with auxiliary phosphine **26** (Figure 4) may inhibit Top1 by blocking the relegation step and inhibit Top2α by preventing ATP hydrolysis, as proposed by molecular docking analysis. They also perturb DNA replication, generate ROS, and induce apoptosis [113].

## 3. Cell Cycle Regulation by Copper Complexes and Top Inhibitors

CuC inhibitors targeting Top1 [72,90] or Top2 [109] as DNA-damaging drugs or poisons arrest cancer cells in G2/M (Table 1). This common G2/M arrest involves the activation of two different cell cycle pathways: the DNA damage response (DDR) and the decatenation checkpoint.

**Figure 5:**
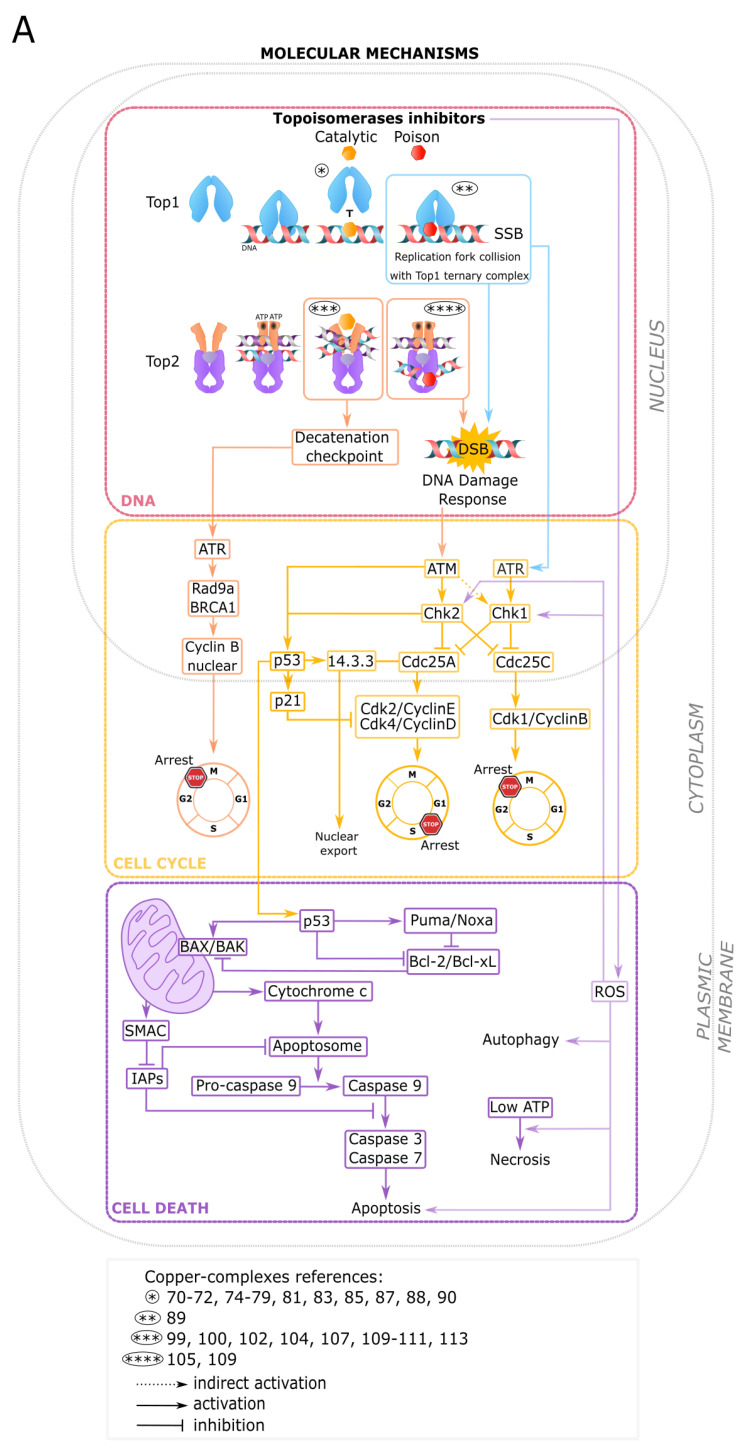

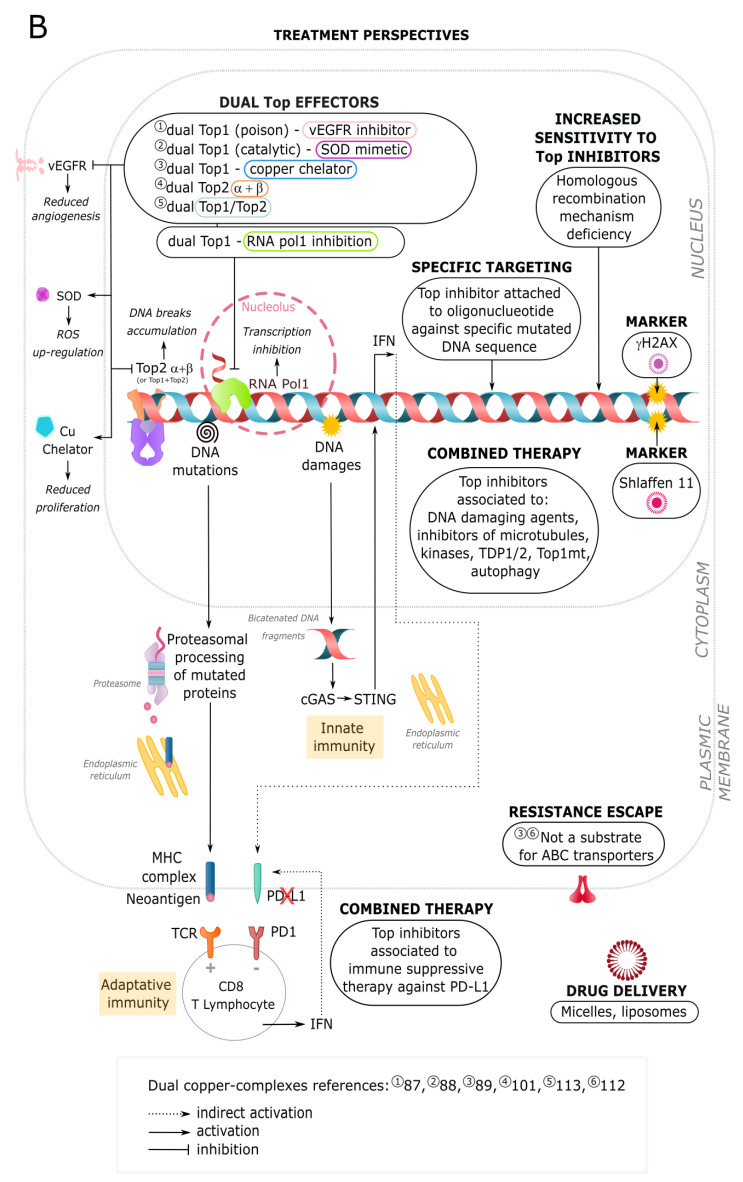
Molecular mechanisms and treatment perspectives for copper complexes (CuC) drugs. (**A**) Molecular checkpoints and networks involved in DNA damage (red), cell cycle regulation (yellow), and death response (violet) triggered by topoisomerase inhibitors (poison and catalytic), including CuC of topoisomerase inhibitors. (**B**) Treatment perspectives alone or in association with other chemotherapeutics (see text for more details).

Both Top1 and Top2 CuC inhibitors produce DNA damages. Top2 poisons prevent DNA relegation and stabilize an enzyme–DNA complex with the double-stranded cleaved DNA [114]. Top1 poisons induce single-stranded DNA breaks and associated signaling cascades. The collision between the Top1 cleavage complexes and the DNA replication forks ends up generating double-strand breaks [115] (Figure 5A). Top1- and Top2-induced DNA breaks trigger a DDR executed by ATM-, ATR-, and DNA-PK-related kinases, and an arrest of the cell cycle machinery [116,117,118]. ATM- and ATR-dependent phosphorylations of p53, Chk1, and Chk2 regulate the G1/S, S, or G2/M cell cycle checkpoints. Chk1 and Chk2 inhibit Cdc25 phosphatases (A,B,C) required for Cdks activation. Phosphorylated and ubiquitinated Cdc25A (Ser123) is degraded, leading to the absence of activation of the Cdk2/Cyclin E and the Cdk4/cyclin D complexes and followed by an arrest in G1/S. Phosphorylated Cdc25C (Ser216) binds to 14-3-3, prevents Cdk1/Cyclin B (MPF) activation, and induces a G2/M arrest (Figure 5A). Cdc25B inactivation also results in a G2 arrest [119,120]. The DNA damage-induced cell cycle arrest in G1 is dependent on p53 phosphorylation by ATM (Ser15) and Chk2 (Ser20) but arrest in S and G2 phases is p53-independent [121,122,123,124]. Phosphorylated p53 dissociates from MDM2 and activates the transcription of Cdk inhibitor p21WAF1 [125,126]. In several CuC (Top1 DNA binding CuC inhibitors [72,82,88] and a dual Top1/2 inhibitor with heteroleptic CuC [113]), Cu(II) exhibits a high redox potential and reinforces DDR activation by ROS production. ROS are also involved in a G2/M arrest through the decrease in Cdc25C [127] and Cdc25A levels [128], the activation of Chk1 [129] and Chk2 [130], and genomic instability through induced-DNA damages [131] (Figure 5A).

By contrast to poisons, Top2 catalytic inhibitors do not form cleavable complexes. They function by enzymatic activity deprivation and cell cycle arrest in G2 through a decatenation checkpoint distinct from the DNA damage checkpoint. To delay the mitotic entry, an insufficient decatenation engages molecular components from the DDR and the spindle assembly checkpoint (SAC) (Rad9a, ATR, and BRCA1), SUMOylation and phosphorylation of Top2, the p38 and the MAPK pathways, and several decatenation checkpoint effectors but not p53 [66,132,133,134,135,136] (Figure 5A).

Cell cycle checkpoint effectors arrest DNA-damaged cells and induce their death providing that cell cycle regulatory networks are effective. Cell cycle checkpoint effectors integrity influences responses to Top2 inhibitors [137]. Besides, cancer disease is associated with multiple overexpression and mutations [138] in Cdc25 [139,140] and p53 [141,142], to a loss of Cdk inhibitors expression and/or overexpression of cell cycle-regulated protein [143,144], Top deregulation, and multidrug resistance [145,146,147]. Moreover, cell cycle variation of Top2α is regulated by post-translational modifications that represent potential targets. These alterations include ubiquitination by Cdk-1 [148], sumoylation [149], phosphorylation by polo-like kinase 1, Cdc7 [150], protein kinase C, Ca/calmodulin-dependent kinase II, and casein kinase [151], and the association with 14-3-3 [152]. Rewiring cellular pathways leading to cell death is a challenge that requires targeting specific molecular checkpoint effectors [153]. For example, a mutated p53 pathway arrests the cell cycle but avoids DDR-induced cell death [154]. Some anticancer therapeutic strategies (e.g., Chk1/2 pathways targeting drugs associated with DNA-damaging drugs) can force cancer cells to bypass S and G2/M arrest, enter mitosis with damaged DNA, and finally undergo a mitotic catastrophe and death [155]. ATR inhibition is another strategy to overcome the resistance of BRCA-deficient cancers [156].

## 4. Programmed Cell Death Engaged by Copper Complexes and Top Inhibitors

Multiple stress factors ranging from various cell damages, ATP levels, and specific pathways (e.g., caspases) determine the type of cell death [157]. Most Top1 CuC inhibitors that interact with DNA [70,76,79,86,87,90], Top1 poison [89], Top2α CuC poison [109], or dual Top1/Top2 inhibitor [113] trigger apoptotic programmed cell death. Genetic damages and oxidative stress activate an intrinsic mitochondrial response [158]. Pro-apoptotic members of the Bcl-2 family (Bid, Noxa, Puma, BAX, BAK) neutralize the anti-apoptotic members (Bcl-2, Bcl-xL, and Mcl-1), disrupt the mitochondrial outer membrane, and allow cytoplasmic cytochrome-c release. The binding of cytochrome c to the apoptotic protease activating factor-1 (Apaf-1), ATP, and the pro-caspase-9 create the apoptosome protein complex. Pro-caspase 9 is cleaved into its active caspase-9 form, which in turn cleaves pro-caspase-3 into caspase-3 effector, and the downstream executor caspase-7. SMAC (second mitochondria-derived activator of caspases), and Omi/HtrA2 (high-temperature requirement protein A2) are simultaneously released from mitochondria and deactivate the IAPs factors (inhibitors of apoptosis proteins). p53, activated by the DNA damage, contributes to apoptosis through the translation of several pro-apoptotic members of the Bcl-2 family (Bid, Puma) that inhibit the pro-survival action of Bcl-2 on BAX (Figure 5A). Most cancer cells evade apoptosis through caspase inhibition, upregulation of Bcl-2 (in more than 50% of all types of cancers), and loss of BAX/BAK and become resistant to anticancer drugs [159].

A Top1 DNA-damaging CuC inhibitor induces necrotic cell death. To facilitate cell destruction, necrosis is activated by ROS or ATP metabolic stresses in crosstalk with apoptosis [160]. When the intracellular energy/ATP level is low, the apoptotic cell death is converted into necrosis [161] (Figure 5A). However, necrosis releases pro-inflammatory and tumor-promoting cytokine HMGB1 [162] into the extracellular space reported to stimulate inflammation and angiogenesis, and promote tumor progression [163].

Apoptosis and necrosis often co-exist with another cell death with controversial pro-death and pro-survival functions: autophagy [164]. Up to the current study, no CuC Top inhibitors are involved in autophagic or necroptotic programmed cell death (Table 1). However, some CuC trigger stress-mediated protective autophagy in response to ROS that impedes apoptosis and creates survival of malignant cells [165]. Moreover, topoisomerase inhibition-induced autophagy is associated with cancer resistance [166].

## 5. Future Strategies for Copper Complexes as Top Inhibitors in Cancer Cell Treatments

The development of new effective anticancer drugs is a major research area against the continuing increase in cancers worldwide. Top inhibitors used in chemotherapy are limited in number [61,167,168]. Top1 inhibitors’ camptothecin derivatives used are irinotecan (colorectal [169], pancreatic (in combination) [170], and small cell lung cancers (in clinical trials and in combination) [171,172]), and topotecan (ovarian [173,174], cervical [175], and small cell lung cancers [176]). Top2 anticancer drugs commonly used are from the anthracycline group such as doxorubicin (acute leukemia [177], lymphomas [178], sarcomas [179,180], and solid tumors [181]), epirubicin (breast cancer [182]), valrubicin (bladder cancer [183]), and idarubicin (acute myeloid leukemia [184]), from the anthracenedione classes: mitoxantron and pixantron (lymphoma, [185,186,187]), and from the epipodopodophyllotoxins group such as etoposide (testicular [188] and small cell lung cancers [189]) and teniposide (brain [190] and small cell lung [191] cancers, acute lymphocytic leukemia [192]). Only a few numbers of Top1 inhibitors are in clinical trials including the promising indenoisoquinoline derivatives LMP400 (Indotecan), LMP776 (Indimitecan) (phase I), and LMP744 examined in a phase I study on lymphoma in dogs [193]. In addition to better stability, and milder side effects, they can escape ABC transporter efflux and the drug resistance mechanism, as Elesclomol-CuC Top complexes **13** [89] or Quinolinone-CuC **25** [112]. Perspectives to use CuC of Top inhibitors in clinical trials are summarized in Figure 5B. Development and optimization in CuC of Top inhibitors imply structure modifications that must encompass several specific strategies [194], such as scaffold hopping [195], pharmacophore hybridization [196], bioisosteric replacement [197], and conformational restrictions. Generally, a rigidification of the ligand heterocycle structure with a copper metal [78] provides a planar configuration that facilitates DNA intercalation and Top-DNA ternary complex formation compared to the molecular backbone alone.

Top inhibitors in clinical use and particularly Top poison display unwanted drawbacks, such as cumulative cardiotoxicity in long-term protocols, secondary malignancies, and drug resistance [198]. A therapeutic option would be to use preferentially catalytic Top agents that disturb the catalytic cycle without the formation of a ternary complex. CuC Top catalytic inhibitors, listed in Table 1, exhibit high antitumor effects on cancer cell lines and for some compounds on tumor growth in animal models, compared to their respective ligands (see Table 1). They constitute a reservoir of anticancer drugs. For example, TSC-based CuC Top2 inhibitors (Figure 3) [98,102,103,105,107] have demonstrated strong inhibition of tumor growth compared to TSC derivatives currently used in cancer chemotherapies [199].

Considering that cancer is a multigenetic and multifactorial disease that recruits numerous molecular effectors, monotherapies (based on Top inhibitors) do not provide the optimal curative effects. Combination therapy with a few numbers of therapeutics against two or more biotargets is the base of promising treatments such as the association of a Top 2 inhibitor (vosaroxin) with a DNA methyltransferase inhibitor (decitabine) in AML [200,201]. Inhibitors of Top1 and Top2, currently developed, also exert their effect against other cancer-related targets [202]. Dual Top inhibitors, e.g., Top 1/2 [203], Top2/microtubule [204], or Top2/histone deacetylase [205], may exert improved efficacy. Besides, Top1 inhibitors are nonspecific RNA polymerase inhibitors. An RNA Pol1-mediated ribosomal RNA gene increase is involved in cancer progression, through the control of cellular checkpoints and chromatin structure and is, therefore, an interesting co-target [206]. CuC dual Top inhibitors display a high antiproliferative activity. Particularly, some CuC and non-CuC are dual inhibitors of Top1 and superoxide dismutase agonist [88,207,208] or Cdk receptor, like VEGF inhibitors, involved in cancer cells proliferation [86,87,209,210] (Figure 5B). Another strategy to improve therapies is the association of a CuC with a TDP1/2 (tyrosyl-DNA-phosphodiesterase 1/2) inhibitor. TDP1/2 are enzymes responsible for the reparation of DNA breaks induced by topoisomerase poisons [57,211,212]. TDP1/2 inhibitors are capable of improving cancer cells’ sensitivity to these poisons [213].

Autophagy, an essential mechanism for cell integrity and survival, is stimulated in cancer cells under several chemotherapeutic drugs and acts as an unwanted protective system towards tumor cells. Association of specific autophagic inhibitors with Cu-C treatment (disulfiram) in non-small cell lung cancer [214] has proven to be a novel efficient strategy to enhance apoptosis in cancer therapy.

Immunogenic cell death is an important mechanism used in chemotherapy. Association of CuC with immune checkpoint therapies is certainly a new avenue in cancer treatment. CuC and non-CuC Top inhibitors induce DNA damages and are linked to adaptive and innate immunities [215]. Top poisons promote immunogenicity in various ways [216]. Top1 poison camptothecin enhances the adaptive immune response [217]. Top inhibitors also increase chromosomal instability and mutations accumulated by cancer cells [59,218]. Consequently, due to their high number of mutations, tumors display more neoantigens presented at their surface by the major histocompatibility complex class I (MHCI) and recruit lymphocytes T harboring TCR (T cell receptor) and CD8 co-receptor (adaptive immunity). This response is counterbalanced by the overexpression of immune checkpoint modulators, such as the immune-suppressive ligand PD-L1 (programmed death-ligand 1) targeted in immune therapies [219] (Figure 5B). DNA-damaging agents such as Top2 poison anthracycline also interfere with the innate immune response. They enhance the malignant formation of cytosolic bicatenated DNA fragments that activate the cyclic GMP-AMP synthase-stimulator of the interferon (IFN) gene pathway (cGAS-STING) and initiate innate anti-cancer immunity. cGAS-STING agonist serves as a sensitizer in immunotherapies [220]. Top1-DNA covalent cleavage complex enables cGAS-mediated cytoplasmic chromatin recognition and immune checkpoint response [221] (Figure 5B). Top2 inhibitors teniposide and doxorubicin potentiate the therapeutic immune checkpoint blockade therapies based on anti-PD-1 (programmed cell death 1) in multiple types of mouse tumor models [222,223]. Besides, ROS produced by Top inhibitors alter the molecular pattern recognized as immunogenic structures and enhance apoptosis [224] (Figure 5B).

As DDR gene mutations exist in a large range of tumor types, the determination of tumor-specific mutations is another accurate strategy to generate chemotypes with beneficial efficacies superior to adverse effects [225,226]. In each tumor, the signaling components of the DDR exhibit numerous defects that result in a unique mutational signature [227]. Cancer cells with defects in their homologous recombination mechanism are more sensitive to Top2 inhibitory therapies that generate DNA double-strand breaks [228]. Moreover, the prediction of anticancer treatments determined by the clinical stage and the pathological features of the tumor does not always ascertain a cancer death response. Cellular biomarkers that may predict sensitivity or resistance to therapy based on DNA damage induced by Top inhibitors would be useful. Insights into the Top2 regulatory mechanisms have identified genetic markers to allow the prediction of an overcome treatment with a Top inhibitor. γ-H2AX is a DNA-damaged marker, recruited on DNA breaks after Top poison action, currently evaluated [229]. Schlaffen is also a promising marker for an accurate response to Top1 and Top2 inhibitors, especially for colon and ovarian adenocarcinomas [56,230] (Figure 5B).

Recently, cancer cells were targeted specifically by a Top2 inhibitor, etoposide, attached to a single-stranded oligonucleotide with a complementary sequence to a DNA cleavage hotspot corresponding to a translocated region only present in promyelocytic leukemia cells [231].

Finally, to overcome toxicity to normal cells, Top drugs could be attached to vehicles. Top2 inhibitors delivery has been optimized using liposomes [232], micelles [233], or functionalized nanoparticles [234] (Figure 5B).

Topoisomerases are present in mitochondria where they participate in mitochondrial DNA replication and transcription. Mitochondrial Top1 isoform (Top1mt) is involved in the metabolism of cancer cells providing energy to tumors surrounded by a nutrient-low microenvironment. Exposures to a Top1 inhibitor (lamellarin D) or Top2 inhibitors (doxorubicin or fluoroquinolones) exert mitochondrial toxicity [235]. However, the loss of Top1mt in liver cancers correlates with increased survival of hepatocellular carcinoma patients, showing that co-targeting Top1mt in addition to nuclear topoisomerases is another option for anticancer therapies [236].

## 6. Conclusions

In a multifactorial disease such as cancer, Top inhibitors are efficient anticancer compounds used in monotherapy or polypharmacological strategies. They certainly have to target closely related modulators of the cellular checkpoints’ networks. CuC Top inhibitors are particularly adapted to fulfill this role. A perspective in anticancer strategy is to increase and to enlarge this family of highly active anticancer drugs.

## Figures and Tables

**Table 1:** Copper complexes inhibitors of topoisomerases: targeted top isoforms, cancer cell lines responses, and molecular mechanisms are summarized. * Tests were realized in vitro with human Top1 or Top2α/β unless specified. IC50: half-maximal inhibitory concentration. EC50: half-maximal effective concentration. GI50: half-average of growth inhibition.

Ligand Class of Cu-C	Compound Number	Targeted Top(s)	Inhibition of DNA Relaxation Total (µM) (minimal (µM))	Inhibition Mecanism	Cancer Cell Lines	IC50 (µM)	Cell Cycle Arrest	Cell Death Type	Other Specificity	Reference Number
Oxindolimine	**1**	Top1	50 (25)	Fixation in the DNA Top1 binding site	Neuroblastoma SH-SY5Y Promonocytic U937		G2/M arrest	Apoptosis	ROS induction	[70,71,72,73]
			
Hydrazone with triphenylphosphonium	**2**	Top1	40	DNA Binding	Lung A549	4.2 ± 0.8				[74]
				Enzyme complex formation	Prostatic PC-3	3.2 ± 0.2				
Plumbagin	**3**	Top1	1.56	DNA intercalation	Breast MCF-7	3.2 ± 1.1				[75]
					Colon HCT116	5.9 ± 1.4				
					Hepatoma BEL7404	12.9 ± 3.6				
					Hepatoma HepG2	9.0 ± 0.7				
					Kidney 786-O	2.5 ± 0.9				
					Lung NCI-H460	2.0 ± 1.2				
					Nasopharyngeal cancer CNE2	11.8 ± 5.9				
Phenanthroline with amino acids	**4**	Top1	50	DNA intercalation	Nasopharyngeal cancer HK1	2.2–5.2		Apoptosis		[76]
			(10)							
Pyrophosphate	**5**	Top1	500	DNA interaction	Ovarian A2780/AD	0.64 ± 0.12				[77]
Heterobimetallic Cu(II)-Sn2(IV) phenanthroline	**6**	Top1	20	DNA intercalation	Breast Zr-75–1					[78]
				cleavage	Cervix SiHa					
					Colon HCT15, SW620	<10 (GI50)				
					Kidney 786-O, A498					
					Lung Hop-62, A569					
					Pancreatic MIA PaCa-2					
					Neuroblastoma SH-SY5Y	2–8		Apoptosis		[79]
Analogs										[80]
Tridentate chiral Schiff base	**7**, **8**	Top1	25	DNA binding	Hepatoma HuH7	25			ROS	[81,82]
			(15)	major groove	Hepatoma HepG2	6.2 ± 10			Cytokine TGFb	
									mRNA upregulation	
Salicylidene	**9**	Top1	(*E. coli*) *	DNA binding	Prostatic PC-3	7.3 ± 0.2			antimetastasis	[83]
				DNA cleavage	Breast MCF7	51.1 ± 1.6				[84]
					Colon HT29	16.6 ± 0.6				
					Hepatoma HepG2	2.3 ± 0.1				
					Lung A549	16.8 ± 1.0				
					Ovary A2780	14.6 ± 0.2				
					Prostatic LNCaP	25.4 ± 0.8				
Chalcone-derived Thiosemicarbazone	**10**	Top1	3	DNA binding	Breast MCF-7	0.16 ± 0.06				[85]
			(0.75)	DNA cleavage	Leukemia THP-1	0.20 ± 0.06				
				Religation inhibition						
Pyridyl-substituted tetrazolopyrimidie	**11**	Top1	(Molecular docking) *	DNA binding	Cervix HeLa	0.565 ± 0.01		Apoptosis	CDK receptor	[86]
				groove mode	Colon HCT-15	0.358			binding	
					Lung A549	0.733				
Tetrazolopyrimidine Diimine		Top1	102 ± 1.1	DNA binding	Cervical HeLa	0.620 ± 0.0013		Apoptosis	vEGF receptor	[87]
				groove mode	Colon HCT-15	0.540 ± 0.00015			binding	
					Lung A549	0.120 ± 0.002				
Piperazine	**12**	Top1	12.5	DNA binding					SOD mimic	[88]
			(5)	minor groove						
Elesclomol	**13**	Top1	50	Poison	Erythroleukemic K562	0.0075		Apoptosis	Copper chelator	[89]
								Necrosis	Not a substrat for	
								Oxidative stress	ABC transporters	
Cu(SBCM)2	**14**	Top1	* (Molecular	DNA intercalation	Breast MCF7	27	G2/M arrest	Apoptosis	p53 increase	[90]
			docking)	DNA binding	Breast MDA-MB-231	18.7 ± 3.1			No ROS	[91]
TSC and TSC CuC										[92,93,94,95,96,97]
Pyridine-TSC	**15**	Top2a	50		Breast MDA-MB-231	1.01				[98]
			(10)		Breast MCF7	0.0558				
			50	ATP hydrolysis inhibition						[99]
		Top2β	(5)	ATP hydrolysis inhibition						[100]
Piperazine-TSC	**16**	Top2a	0.9 ± 0.7	Potentially catalytic	Breast MCF7	4.7 ± 0.3				[101,102]
					Breast SK-BR-3	1.3 ± 0.3				[99]
Thiazole-TSC	**17 **	Top2a	4		Breast MDA-MB-231	1.41 (EC50)				[103]
			(2)		Breast MCF7	0.13 (EC50)				
	**17–18**	Top2a	25	ATP hydrolysis inhibition	Breast					[104,105]
			(10)	+ Poison	HCC 70, HCC 1395,	1 to 20				
					HCC 1500, and HCC 1806					
					Colon	0.83 to 41.2				
					Caco-2, HCT-116 and HT-29					
L- and D-Proline-TSC	**19**	Top2a	300		Ovarian carcinoma CH1	113 ± 16				[106]
Quinoline-TSC	**20**	Top2a	0.48	Potentially catalytic	Lymphoma U937	0.48-16.2				[107]
Naphthoquinone-TSC	**21**	Top2α	1 mM		Breast MCF7	3.98 ± 1.01		No apoptosis		[108]
Bis-TSC	**22**	Top2a	100	Poison	Breast MDA-MB-231	1.45 ± 0.07	G2/M arrest	Apoptosis	DNA synthesis	[109]
			(5)		Colon HCT116	1.23 ± 0.27			inhibition	
					Keratinocyte HaCaT	0.65 ± 0.07			No ROS	
					Colon HCT116	Delayed mice xenograft				
Carbohydrazone	**23**	Top2α	250	DNA binding	Breast MCF7	9.916		Apoptosis		[110]
			(25)	major groove	Breast MDA-MB-231	7.557				
					Breast HCC 1937	3.278				
					Breast MX1	4.534				
					Breast MDA-MB-436	5.249				
					Breast MX-1	Reducted mice xenograft (83%)				
Chromone	**24**	Top2a	25	DNA binding	Breast MCF7	18.6 (GI 50)				[111]
			(15)	major groove	Breast Zr-75-1	25.2 (GI 50)				
					Colon HT29	>80 (GI 50)				
					Cervix SiHa	34.6 (GI 50)				
					Kidney A498	73.3 (GI 50)				
					Lung A549	31.7 (GI 50)				
					Ovary A2780	17.4 (GI 50)				
Quinolinone Shiff Base	**25**	Top2α	9	No intercalation	Hepatic HepG2	17.9 ± 3.8			DNA synthesis	[112]
									inhibition	
									Slight substrate	
									for ABC transporter	
Bis-pyrazolyl Carboxylate	**26**	Dual Top1/Top2	(Molecular docking) *	ATP entry (potentially)	Hepatic HepG2	3.3 ± 0.02		Apoptosis	DNA replication	[113]
				DNA religation inhibition (potentially)					ROS	
